# Comparison of patient satisfaction with services of Vision Centers in rural areas of Andhra Pradesh, India

**DOI:** 10.4103/0301-4738.67056

**Published:** 2010

**Authors:** Vilas Kovai, Gullapalli N Rao, Brien Holden, Krishnaiah Sannapaneni, Shubhra K Bhattacharya, Rohit Khanna

**Affiliations:** 1 International Centre for Advancement of Rural Eye Care – LV Prasad Eye Institute, Hyderabad, India; 2 Vision Cooperative Research Centre Pty Ltd, Sydney, Australia; 3 International Centre for Eye Care Education, Sydney, Australia; 4 Institute for Eye Research, Sydney, Australia

**Keywords:** Large village (s), small village (s) and refractive services, patient satisfaction, vision center

## Abstract

**Aim:**

To compare the satisfaction of patients with the services of Vision Center services (primary eye care) in large village (s) and small village (s) in rural settings in Andhra Pradesh state, India.

**Materials and Methods:**

We have administered standard questionnaires to randomly selected patients to assess patient satisfaction when assessing Vision Center Services. We used the Chi-square (*P* <0.05) to explore differences in satisfaction of patients with Vision Center services located in the large village (s) and small village (s) rural settings.

**Results:**

Vision Center patients at the large village (s) expressed higher levels of satisfaction (median 78%) than patients treated at the Vision Center at small village (s) (median 69%). The difference was statistically significant (Chi square *P* value ranging from <0.001 to 0.03) for all the items except two – ‘easy to identify vision center location’ and ‘spectacles dispensing time’ as compared to other (privately run optometry) facilities (Chi square *P* value=0.498 and 0.993 respectively). The location of the Vision Center, convenience of journey, ophthalmic technician’s behavior with patients, are some of the most important factors that determined the patient perception about Vision Center services.

**Conclusion:**

The overall satisfaction levels of the Vision Center experience at 78% and 69% were good. However, continual improvement is to be made in service time, staff performance, cost and quality of vision care, especially at more remote primary eye Care Centers.

Rural populations, worldwide, have poor access to eye care services leading to sub optimal utilization of existing services.[1–5] The five important dimensionsof access include - availability, accessibility, accommodation, affordability and acceptability.[6] Affordability and availability, besides accessibility, of eye care services are major barriers to utilization of eye care services in India.[7–18]

An analysis of causes of avoidable blindness highlights the fact that a properly planned and developed system of primary eye care can have greater impact by increasing accessibility, availability and affordability of eye care to the underserved populations of the world.

To this end, in India, we find several relatively novel programs that aim to provide accessible and affordable eye care to marginalized populations.[19] “Vision Center” is one such program, (developed by L V Prasad Eye Institute, Hyderabad, India, in 2002) designed to address the correction of refractive errors, (the major cause of visual impairment and blindness) detecting cataract and other potentially blinding conditions, health education, and appropriate referrals to the secondary eye care center. These Vision Centers are located closer (compared to privately run optometry services) to the community, either in small towns or in villages.

Relevant literature review indicates that patient satisfaction plays major role in the acceptance of health care services.[20–24] Although the Vision Center is now considered an integral part of the VISION 2020: The Right to Sight Initiative, little evidence exists on the satisfaction of the target community with the services provided; which is also a key measure of healthcare quality.

We tested the hypothesis that patients accessing the services of Vision Center in large Village (s) would be more satisfied primarily due to ease and lower cost of accessing services compared with access to Vision Center in small Village (s).

## Materials and Methods

We used retrospective and comparative study designs and structured and unstructured questionnaires to obtain data from 136 randomly selected patients on patient satisfaction. The data was collected from October to December 2007 among patients attending four Vision Centers, two easily accessible to the rural population they served (large village (s)/hub and two serving patients from remote areas (Small village (s)/hub. These Centers were randomly selected (simple random selection without replacement from a sample frame that included all established Vision Center) to represent the large village (s) and small village (s) context of the Vision Center Project [Table 1].

**Table 1:** Criteria^a^ used for selection of sampled Vision Centers (VCs) in large village (s) and small village (s)

^c^Criteria for Selection of Vision Centers	1	2	3	4	1	2	3	4

Different geographical settings	Large villages	Large villages	Large villages	Large villages	Small villages	Small villages	Small villages	Small villages
Over all skills of Ophthalmic Technician*	4	3	4	3	3	2	2	3
Performance of Vision Center (patient volumes)	4	3	3	4	2	1	1	2
Financial sustenance of Vision Center**	4	3	3	4	2	2	1	2
(Cost recovery met through spectacle sales)								
Community perceptions about Vision Center**	3	4	2	3	2	3	1	1
Provider cost per patient at Vision Center**	4	3	4	3	2	2	1	1
Involvement of Ophthalmic Technicians to improve the performance of Vision Centers**	4	2	2	3	3	2	2	1

a Criteria were rated on: 0 (very dissatisfied) to 4 (very satisfied), 0 =Not at all, 1 = poor, 2 = moderate, 3 = good, 4 = excellent

* Rated by author, optometrist and social scientist as part of periodical monitoring and evaluation of program

** Rated by the author using the data from the periodical monitoring and program evaluation, The ratings indicates that on the whole the Vision Centers established in large villages, can be grouped in to one category and Vision Centers of small villages, Can be grouped in to another category.

In this study, we often use three terms namely – A) large village (s), B) and small village (s), and C) patient satisfaction and we defined these terms as follows:

- Large village (s): It is a hub with a base population of approximately 20,000 people, with well developed market, educational facilities and connected to other small hubs and villages.
- Small village (s): It is a remote hub with a base population less than 5,000 people, without basic facilities such as market, education and transportation and connected to other small villages.
- Patient satisfaction: We define patient satisfaction as ‘positive evaluations of specific aspects of healthcare facility.’[20] In our context it refers to a measurement that obtains ratings from patients about services received from Vision Center similar to what was reported in the literature.[21–24]

The Vision Center Concept was to provide a model of “primary eye care” in the remote and underserved geographical areas of developing countries in a fairly focused approach to 50,000 people and a group of 10 such Vision Centers is attached to one Secondary Care Center. The initial capital cost to develop the infrastructure is less than 15 Indian rupees per person for a population unit of 50,000, which is sourced through external funding (Rs. 6.5 - 7.5 lakh) agencies, and the recurrent cost are managed through the sale of low cost spectacles.

One ‘Vision Technician’ staffs each Vision Centre. Potential technicians are selected from the community in which they will serve and must have a minimum education of high school diploma. Vision Technician training requires one year and includes refraction assessment, slit lamp evaluation, and applanation tonometry. Vision technicians are trained to handle three primary eye care functions: a) recognizing the patients eye problem, b) performing appropriate refraction and dispensing spectacles and c) educating and referring the patients with potential blinding conditions to secondary eye care centers for medical and surgical management.

As on 2006, there are 30 functioning Vision Center Centers in the underserved areas of state of Andhra Pradesh, India. Of these 30 Centers, four have been functioning at large village (s) and four at small village (s) for more than 24 months. All the Vision Centers situated at the large village (s) have been successful in achieving the objectives of the Vision Center model in terms of patient volumes (18 -20 patients per day) and are financially self reliant as compared to the Vision Centers situated in small village (s) (six to eight patients per day)

The screening and refraction services are free of cost and charge for spectacles is the same regardless of the Vision Center location, so the choice between locations for Vision Center services could reasonably be influenced by questions of travel convenience, effectiveness and patient preference. In this study, we employed a combination of qualitative (case study approach with participatory techniques) and quantitative approaches to understand the difference in levels of satisfaction with Vision Center services in the two different settings and identify the factors that contribute to this variation.

**Figure 1:**
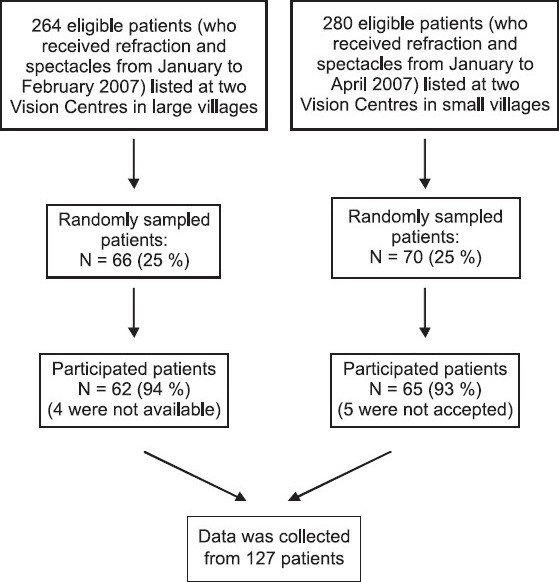
Selection of sample for the study

Using our field experience and qualitative data on Vision Centers, we presumed that there would be a minimum of 10% difference in the effectiveness of service delivery of the Vision Centers of large villages over the Vision Centers of small villages (with a margin of error at 5%). Accordingly, a sample of 136 subjects was needed in this study. To obtain 136 eligible patients, we sampled every 4^th^ patient on the list with the starting point for the sample being randomly selected from the list of the first four patients [Fig. 1].

The selection criteria for the 136 sampled patients included: (a) patients above 16 years who received eye screening and spectacles at least six months prior to the study (b) patients who lived within 10 km distance from the Vision Center locations. The ethics committee of L V Prasad Eye Institute approved this study. We obtained informed consent of the participated subjects after explanation of the nature and possible consequences of the study.

We followed a multi-step procedure at three stages for developing the questionnaire used in this study. Stage 1 consisted of reviewing the relevant literature on eye care, observing the Vision Center services and documenting experiences from patients and staff to understand the appropriate themes of the study questionnaire. Stage 2 entailed interaction with social scientists, public health expertise, eye care managers and Ophthalmologists to discuss the wording, content, relevance and number of items to be included in the questionnaire. During this stage, we have developed 30 case studies from two types of Vision Center settings using qualitative methods. At stage 3, questions were designed using the themes developed from the initial 30 case studies on the selected Vision Centers as well as reviewing the Group Health Association of America consumer Satisfaction Survey[25] and Lothian Health Survey[26] (These questionnaires are used in the published literature and suited Vision center context). At stage 3, the questionnaire (N = 16 items) was piloted on 60 subjects. We asked the patients “How satisfied or dissatisfied with various aspects of Vision Center services”? We assessed the responses on 5 – point Likert scale from 0 (very dissatisfied) to 4 (very satisfied).

Two trained field investigators (Masters of social work) with an interval of one week piloted the questionnaire independently. (The investigators were not aware of the details of the data collected by their counterpart). The principle investigator who was not part of the administration of the questionnaire compiled the data for analysis. We used Cohen’s kappa statistic method to find the agreement between two investigators for each item used to assess patient satisfaction with Vision Center services and the agreement was found to be satisfactory. The kappa values ranged from 0.71 to 0.87 for all the items excepting two items quality (0.41) and affordability of Vision Center services (0.46). The questionnaire was then modified to suit the context of Vision Center based eye care services. The Cronbach’s α statistic was calculated to determine the internal consistency of the 12 questionnaire items to collect satisfaction data (Cronbach’s α = 0.767 for 12 items). After the pilot, the content and number of questions of the questionnaire was modified and the final questionnaire had 12 items. We also used the concept of redundancy as a measure to determine the content of the questionnaire. We used the Pearson’s Chi-square (χ^2^ ) test (*P*<0.05) to explore differences in satisfaction of patients with Vision Center services located in the large village (s) and small village (s).

## Results

A total of 62 patients from the two Vision Centers at large village (s) and 65 patients from the two Vision Centers at small village (s) were interviewed. The total participation rate was 94% in the Vision Centers – large village (s) sample (4 were not available) and 93% in the Vision Centers – small village (s) sample (5 refused to participate). Characteristics of the two samples are compared in Table 2.

**Table 2:** Characteristics of sample in Vision Centers (N = 127)

Characteristics	Two Vision Centers in large villages^a^ (N = 62*)	Two Vision Centers in small villages^b^ (N = 65**)
Refraction and Spectacle received	62 (100.0)	65 (100.0)
Sex: Female	29 (46.7)	31 (47.6)
Age: 60 and above	23 (37.0)	18 (27.6)
16 – 59	39 (62.9)	47 (72.3)
Laborers	44 (70.9)	52 (80.0)
Others	18 (29.0)	13 (20.0)
Literates	17 (27.4)	13 (20.0)
Illiterates	45 (72.5)	52 (80.0)

a Vision Centers located in large villages (s) (famous and big hub) in Andhra Pradesh, India

b Vision Centers located in small village (s) (Remote and small hub) with in Andhra Pradesh, India

* Sampled patients accessed services of VCs of large villages from January to March 2007-2008

** Sampled patients accessed services of VCs of small villages from January to May 2007-2008, Figures in parentheses are in percentage

The number of females was almost equal to the number of males in both samples. The mean ages were 51.1 ±8.7 for large village (s) – rural setting and 49.9 10.4 for small village (s)– rural setting and was not statistically different (*P* = 0.625).

Table 3 gives the percentages of “poor, moderate and excellent” responses in each of the two samples. In all cases the Vision Center patients at large village (s) expressed significantly higher levels of satisfaction than the small village (s) Vision Center patients and the difference was statistically significant for all the items except two (*P* values ranged <0.001 – 0.03). The exception was when patients were asked- i) whether it was easy to identify the Vision Center location and ii) whether the spectacle dispensing time at Vision Center Centers was lower as compared to other facilities in the same region was perceived to be not significantly different. (*P* = 0.498 and 0.993). [Table 4] gives the mean scores of satisfaction for “poor, moderate and excellent” responses in each of the two samples. The final questionnaire was presented as an Appendix.

**Table 3:** Patient satisfaction about Vision Center services in rural settings^a^

Characteristics of Vision Center services (N =12)	Two Vision Center services in large villages (s) (N =62)			Two Vision Center services in small village (s) (N = 65)				df
	0-1	2	3- 4	0-1	2	3- 4	*P*	
Transport convenience	9 (14.5)	26 (41.9)	27 (43.5)	44 (67.7)	13 (20.0)	8 (12.3)	< 0.001*	2
Easy to identify VC	10 (16.1)	10 (16.1)	42 (67.7)	6 (9.2)	12 (18.5)	47 (72.3)	0.50	2
Working hours	0 (0)	10 (16.1)	52 (83.9)	8 (12.3)	15 (23.1)	43 (66.2)	0.012*	2
Waiting room facility	18 (29.0)	34 (54.8)	10 (16.1)	1 (1.5)	11 (27.7)	46 (70.8)	< 0.0001*	2
Time spent by Vision Technician with patient at Vision Center	0 (0)	0 (0)	62 (100)	10 (15.4)	6 (9.20)	49 (75.4)	0.005*	2
Information and guidance	0 (0)	2 (3.2)	60 (96.8)	0 (0)	20 (30.8)	45 (69.2)	< 0.0001*	1
Over all behavior with patient	0 (0)	2 (3.2)	60 (96.8)	6 (9.20)	32 (49.2)	27 (41.5 )	< 0.0001*	2
Importance of Vision Center facility/Value to the beneficiary	0 (0)	8 (12.9)	54 (87.1)	0 (0)	19 (29.2)	46 (70.8 )	0.037*	2
Cost of spectacles	8 (12.9)	25 (40.3)	29 (46.8)	14 (21.5)	36 (55.4)	13 (23.1)	0.018*	2
Quality of Vision Center services to other provider	9 (14.5)	28 (45.2)	25 (40.3)	15 (23.1)	37 (56.9)	13 (20.0 )	0.039*	2
Spectacles dispensing time as compared to other facilities	28 (45.2)	24 (38.7)	10 (16.1)	29 (44.6)	25 (38.5)	11 (16.9 )	0.993	2
Affordability at Vision Center as compared to other facilities near by	8 (12.9)	25 (40.3)	29 (46.8)	16 (24.6)	36 (55.4)	13 (20.0)	0.005*	2

a Patient satisfaction was measured on 0 -1 (lowest) to 3 - 4 (highest scale) using the main question- ‘Overall, how satisfied or dissatisfied are you with the listed characteristics of Vision Center Services’?

* *P* ≤ 0.05, Figures in parentheses are in percentage

**Table 4:** Mean points scored on patient satisfaction

Mean points scored	VCs in large villages				VCs in small villages			
	0- 1	2	3 and 4	%	0 -1	2	3 and 4	%
Transport convenience	4.5	56	87.5	68	7.5	74	45.5	56
Easy to identify VC	14	48	35	45	14.5	50	38.5	45
Working hours	0	0	182	84	4	30	151	80
Waiting room facility	9	68	35	52	0.5	22	161	90
Time spent by Vision Technician	0	0	217	100	5	12	172	83
Information and guidance	0	4	210	99	0	40	158	87
Over all behavior with patient (personal care, thoroughness and competence)	0	4	210	99	3	64	94.5	71
Importance of VC facility/Value to the beneficiary	0	16	189	94	0	38	161	87
Cost of spectacles	4	50	101.5	72	7	72	45.5	56
Quality of VC to other provider	4.5	56	87.5	68	7.5	74	45.5	56
Spectacles Dispensing time as compared to other facilities	14	48	35	45	14.5	50	38.5	45
Affordability at VC as compared to other facilities near by	4	50	101.5	72	7	72	52.5	58
Mean score*	45	368	1610^a^	78^b^	74.5	530	1253^c^	69^d^

Mean score*: = number of respondents in each category of level of satisfaction multiplied by rated level of satisfaction

1610^a^=sum of mean scores for 3 and 4 level of satisfaction for the VCs of large villages

78%^b^= sum of mean scores for each category of rated satisfaction/ total no of respondents* rated level of satisfaction for the VCs of large villages

1253^b^= sum of mean scores for 3 and 4 level of satisfaction for the VCs of small villages

69%^d^= sum of mean scores for each category of rated satisfaction/ total no of respondents* rated level of satisfaction for the VCs of small villages

To test whether the differences in satisfaction were a result of the differences in the characteristics of the two samples, assessments of the visit overall were broken down by age and gender. The mean ages were 51.1±8.7 for large village (s) setting and 49.9 ±10.4 for small village (s) setting were not statistically different (*P* = 0.625). The results show that the level of satisfaction was also not different between males and females (*P* = 0.16 –1.00).

Qualitative data: Patients’ experiences and perceived benefits were unequal at the two locations of Vision Centers. At the Vision Centers in large village (s), 55% said their visit and receipt of spectacles had been highly useful and valuable. 15% patients rated it as useful and a further 12% said it had been a nice feeling, making a total of 82% positive replies. Respondents at the Vision Centers in large village (s) said that the results had been as they expected or better than they expected (82%), and that their daily tasks related to vision had been improved a little (to some extent) or a great deal (95%).

In contrast, 45% of people said their visit to the Vision Centers of small village (s) was highly valuable, 8% of them rated it as having been useful, and 5% of them said it had been a nice feeling (felt good) and little over three fourth of patients said that their daily tasks related to vision had been improved a little or a great deal.

The aspects that received most appreciation (more so in the Vision Centers of large village(s) were thoroughness, carefulness, competence and personal manner of the ophthalmic technicians, and the time spent with the ophthalmic technician.

Patients were given an opportunity to comment on any aspect of their Vision Center visit experience. This was recorded on tape in their language and transcribed into English. In most respects, comments made by patients at the two Vision Centers were indistinguishable. The overwhelming themes were praise and gratitude.

The qualitative data on patient satisfaction indicates that majority of the patients in both locations were least satisfied with three aspects of Vision Center services namely a) ease of identification of the location, b) the waiting time to receive spectacles, and c) the lack of medical treatment.

## Discussion

Patient satisfaction is one of the important components of health service utilization.[27–32] The overall satisfaction levels of the Vision Center experience at 78% and 69% were good. The rated satisfaction about the location of Vision Center, human resources, optical delivery time, and quality of spectacles was relatively low in the case of patients of the Vision Centers at small village (s) as compared to the large village (s), and this perceived difference was found to be statistically significant. (*P* ≤ 0.01 to 0.03).

Forty three percent of the total patients (n = 65) who accessed the Vision Centers at large village (s) were satisfied with the transport convenience Center as compared to 12% patients who accessed the Vision Centers at small village (s). The mean level of satisfaction with the overall behavior of the ophthalmic technician and with the information and guidance received was 99% in the large village (s). The level of satisfaction with the ophthalmic technician and guidance received was lower (87 and 71%) for those who accessed the Vision Centers at small village (s) and this difference was found to be statistically significant. This evidence and qualitative data suggests that the factors – ease of travel and the presence of friendly informative ophthalmic technician greatly influenced the level of satisfaction of patients.

The general atmosphere at the two sites was quite different. The sampled Vision Centers of large village (s) were established in busy and popular hubs and were easy to reach. The sampled Vision Centers in small village (s) were established in remote hubs where the population flow from neighboring villages was relatively less (except on weekly market day). This indicates that the volume of Vision Center services may also be linked to the geographical settings, level of patient awareness, issues of equity etc in addition to patient satisfaction with Vision Center services, which needs to be tested further.

Qualitative data also indicated that the convenience of the journey to Vision Centers at the large village (s) compared with the small village (s) was an obvious source of relief for majority of the people. But a more convenient journey was not perceived as the only benefit, and perhaps it was not the most important benefit. The perceived advantages at Vision Centers of large village (s) included ophthalmic technician who showed more concern, interest and personal support. A similar finding was reported in the study that evaluated patient satisfaction of cataract surgery received in hospital and outreach clinic.[27] Interestingly, although majority of the patients of Vision Centers both at the large village (s) and small village (s) were disappointed with the spectacle-dispensing time (*P* = 0.99), the patient volumes at the Vision Centers of large villages was quite high. Further work needs to be conducted on how to eliminate this common problem. We did not find any significant difference in perceived satisfaction with Vision Center services among the men and women.

Most of the population of rural areas in the developing world can’t access the most basic eye care such as uncorrected refractive errors, because of the absence of infrastructure, trained human resources and affordable optical supply system. Even where the services are available personal costs, distance, inequity, cultural barriers etc prevent people in need from utilizing these services.[4,12–18] Data on perceptions regarding refractive error correction through Vision Center program are essential in developing this essential step in a comprehensive eye care chain and to model the replication of this service in all needy areas.

The value of this study is that it used a mix of qualitative and quantitative techniques and a standardized questionnaire to obtain data on a most critical feature of any successful system of health care - patient satisfaction. Further developments need the research to clarify solutions to the problems identified, in particular attitude of eye care personnel to the most needy of patients, rigorous and constant evaluation of the standard and effectiveness of care and the cost and supply time for spectacles.

## Appendix Final questionnaire on patient satisfaction with Vision Center services

**Table:**

**Please use the following scale to fill the patient responses**								
Very dissatisfied (very unhappy and angry)	0							
Dissatisfied (unhappy)	1							
Neither satisfied/nor dissatisfied	2							
Satisfied (ok)	3							
Very satisfied (extremely happy)	4							
General perceptions of participants Did you feel ease to travel to the Vision Center: Yes / No Was it easy to identify the Vision Center building: Yes /No What is the perceived level of eye problem: Some what blurred/Severely blurred Can you afford the cost of eye care in general: Yes/ No Patient satisfaction questions (structured): Overall, were you satisfied or dissatisfied with the listed characteristics of Vision Center services: Yes No Can’t say Can you rate the over all satisfaction or dissatisfaction of Vision Center services listed below using 0 – 4 scale?

**SN**	**Characteristics**	**Level of Satisfaction**				**Level of Satisfaction**			
		**0 -1**	**2**	**3**	**4**	**0 -1**	**2**	**3**	**4**

1	What was your level of satisfaction with the Transport Convenience to the Village of VC?								
2	Your level of satisfaction regarding ease in identifying the VC building?								
3	How satisfied were you with the working hours of the VC?								
4	How satisfied were you with the waiting room facility of the VC?								
5	How satisfied were you with the amount of time spent with Vision Technician (VT)								
6	How satisfied were you with the received information and Guidance from VT?								
7	How satisfied were you with the over all behavior of the VT (personal care and thoroughness with the job)								
8	How satisfied were you with the importance (value) of VC facility?								
9	How satisfied were you with the cost of spectacles?								
10	How satisfied were you with the quality of VC services compared to other provider?								
11	How satisfied were you with the spectacles dispensing time as compared to similar ophthalmic/optometry/optician facility?								
12	How satisfied were you with the affordability of VC services as compared to similar Ophthalmic/optometry/optician services								
**Unstructured questions**									
1 Can you tell us the reason for satisfaction or dissatisfaction with services?		

2 Can you tell us your opinion about the benefits of spectacles (with emphasis on any improvement in the vision related tasks)	

3 Can you tell us your opinion on the Vision Technician for the following?	
1 Thoroughness:	
2 Carefulness	
3 Personal manner	
